# The genome sequence of the blue-rayed limpet, *Patella pellucida *Linnaeus, 1758

**DOI:** 10.12688/wellcomeopenres.17825.1

**Published:** 2022-04-04

**Authors:** Mara K.N. Lawniczak, Darwin Tree of Life Barcoding collective, Wellcome Sanger Institute Tree of Life programme, Wellcome Sanger Institute Scientific Operations: DNA Pipelines collective, Tree of Life Core Informatics collective, Darwin Tree of Life Consortium

**Affiliations:** 1 Wellcome Sanger Institute, Cambridge, UK

**Keywords:** Patella pellucida, blue-rayed limpet, genome sequence, chromosomal, Mollusca

## Abstract

We present a genome assembly from an individual *Patella pellucida *(the blue-rayed limpet; Mollusca; Gastropoda; Patellidae). The genome sequence is 712 megabases in span. The majority of the assembly (99.85%) is scaffolded into 9 chromosomal pseudomolecules. The mitochondrial genome was assembled and is 14.9 kilobases in length.

## Species taxonomy

Eukaryota; Metazoa; Spiralia; Lophotrochozoa; Mollusca; Gastropoda; Patellogastropoda; Patelloidea; Patellidae; Patella; *Patella pellucida* Linnaeus, 1758 (NCBI:txid88005).

## Background

The blue-rayed limpet is a subtidal species, typically found on macroalgae ( Sá-Pinto *et al.*, 2005). Its range extends from Norway to Portugal ( “Handbook of the Marine Fauna of North-West Europe”, 2017). The scientific name reflects the dish-like shape (Patella) of the translucent (pellucida) shell and the common name describes the striking dashed stripes of iridescent blue down its shell. The underlying features that result in these iridescent blue rays have recently been described ( Li *et al.*, 2015). These authors speculate that the stripes are present to mimic toxic gastropods and ward off predators though this does not appear to have been tested yet. The blue-rayed limpet is a broadcast spawner, releasing one egg at a time that is externally fertilised. Individuals are thought to have a typical lifespan of about a year, with only a small portion of individuals continuing on into a second year ( “Blue-Rayed Limpet (Patella Pellucida)”, 2019).

## Genome sequence report

**Figure 1.**
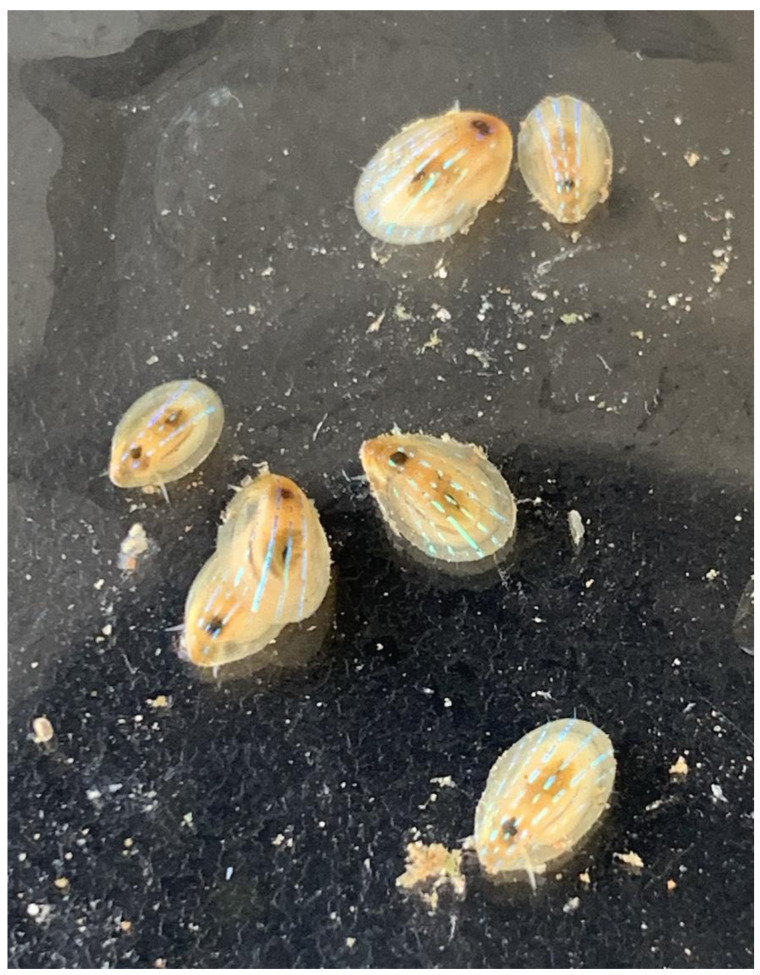
Image of the xgPatPell1, 2, and 3 specimens along with four others not used in long read, long range, or RNAseq data generation for this species’ genome.

The genome was sequenced from a single *P. pellucida* ( Figure 1) collected from Farland Point, Great Cumbrae, North Ayrshire, UK (latitude 55.74629, longitude -4.911721). A total of 31-fold coverage in Pacific Biosciences single-molecule long reads and 55-fold coverage in 10X Genomics read clouds were generated. Primary assembly contigs were scaffolded with chromosome conformation Hi-C data. Manual assembly curation corrected 309 missing/misjoins and removed 29 haplotypic duplications, reducing the assembly size by 1.72% and the scaffold number by 71.56%, and increasing the scaffold N50 by 162.71%.

**Figure 2.**
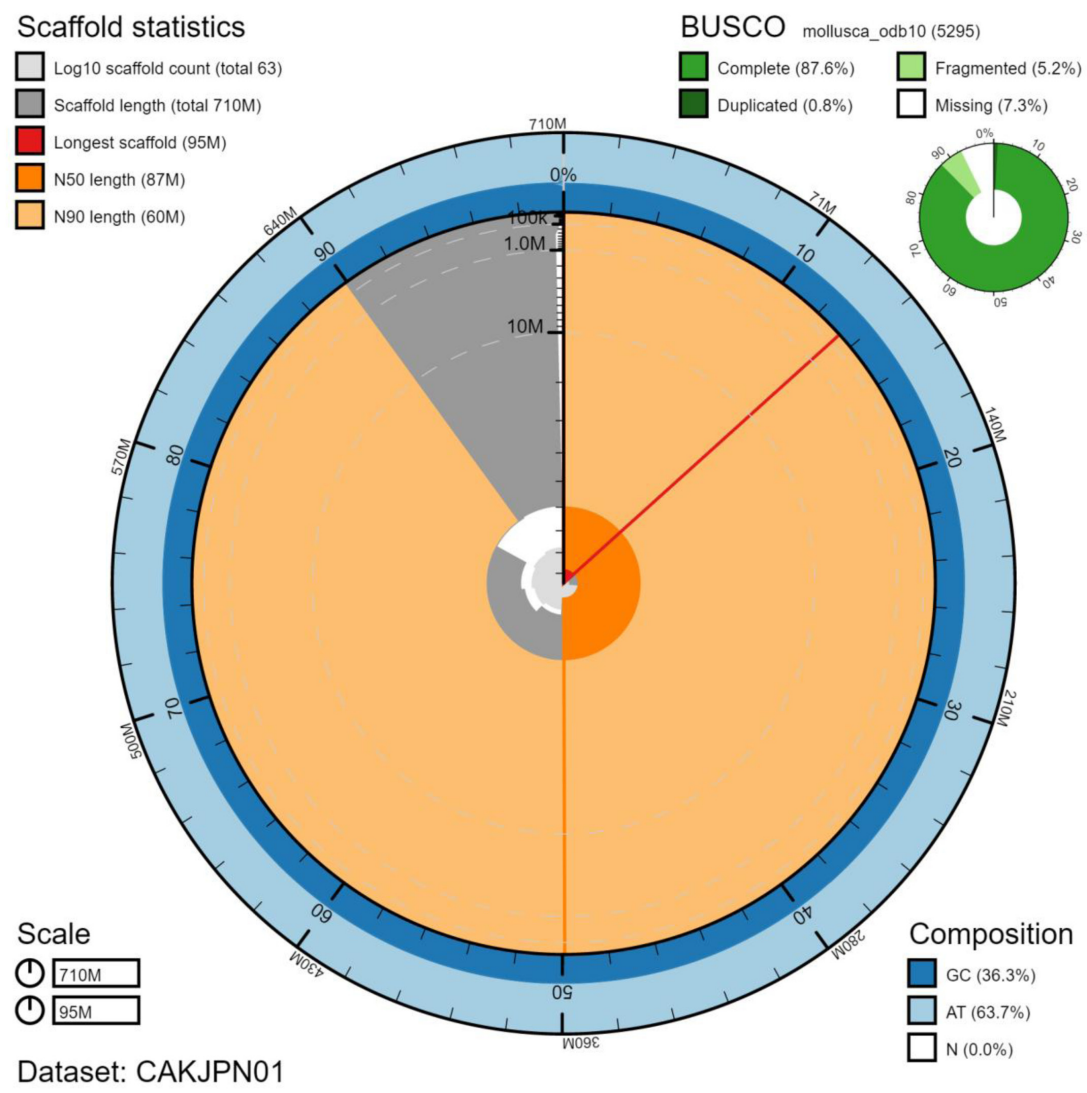
Genome assembly of *Patella pellucida*, xgPatPell1.1: metrics. The BlobToolKit Snailplot shows N50 metrics and BUSCO gene completeness. The main plot is divided into 1,000 size-ordered bins around the circumference with each bin representing 0.1% of the 712,072,003 bp assembly. The distribution of chromosome lengths is shown in dark grey with the plot radius scaled to the longest chromosome present in the assembly (95,029,513 bp, shown in red). Orange and pale-orange arcs show the N50 and N90 chromosome lengths (87,163,620 and 59,729,618 bp), respectively. The pale grey spiral shows the cumulative chromosome count on a log scale with white scale lines showing successive orders of magnitude. The blue and pale-blue area around the outside of the plot shows the distribution of GC, AT and N percentages in the same bins as the inner plot. A summary of complete, fragmented, duplicated and missing BUSCO genes in the mollusca_odb10 set is shown in the top right. An interactive version of this figure is available at https://blobtoolkit.genomehubs.org/view/xgPatPell1.1/dataset/CAKJPN01/snail.

The final assembly has a total length of 712 Mb in 62 sequence scaffolds with a scaffold N50 of 87.2 Mb ( Table 1). Of the assembly sequence, 99.85% was assigned to 9 chromosomal-level scaffolds (numbered by sequence length) ( Figure 2– Figure 5; Table 2). Chromosome 2 contains a large inversion between sister chromatids at approximately 15.43–87.97 Mb. The inversion spans the majority of the 91.75 Mb chromosome. The assembly has a BUSCO v5.1.2 ( Manni *et al.*, 2021) completeness of 87.6% (single 86.7%, duplicated 0.8%) using the mollusca_odb10 reference set (n=5295). However, we believe that this relatively low BUSCO score is a result of limitations with the current mollusca_odb10 geneset. Using the metazoa_odb10 reference set (n=954), the assembly has a completeness of 97.5% (single 96.9%, duplicated 0.6%), which we believe is evidence of high completeness. This value also compares favourably with the 91.5% completeness value obtained by ( Kang *et al.*, 2020) for the transcriptome of related limpet, *Patella vulgata*, using the metazoa_odb reference set. While not fully phased, the assembly deposited is of one haplotype. Contigs corresponding to the second haplotype have also been deposited.

**Table 1.** Genome data for *Patella pellucida*, xgPatPell1.1.

*Project accession data*	
Assembly identifier	xgPatPell1.1
Species	*Patella pellucida*
Specimen	xgPatPell1 (genome assembly); xgPatPell2 (Hi-C); xgPatPell3 (RNA-Seq)
NCBI taxonomy ID	NCBI:txid88005
BioProject	PRJEB45187
BioSample ID	SAMEA7522851
Isolate information	Whole organisms
*Raw data accessions*	
PacificBiosciences SEQUEL II	ERR6412040
10X Genomics Illumina	ERR6054924-ERR6054927
Hi-C Illumina	ERR6054928
PolyA RNA-Seq Illumina	ERR6054929
*Genome assembly*	
Assembly accession	GCA_917208275.1
*Accession of alternate haplotype*	GCA_917208175.1
Span (Mb)	712
Number of contigs	495
Contig N50 length (Mb)	3.2
Number of scaffolds	62
Scaffold N50 length (Mb)	87.2
Longest scaffold (Mb)	95.0
BUSCO * genome score	C:87.6%[S:86.7%,D:0.8%],F:5.2%,M:7.3%,n:5295

*BUSCO scores based on the mollusca_odb10 BUSCO set using v5.1.2. C= complete [S= single copy, D=duplicated], F=fragmented, M=missing, n=number of orthologues in comparison. A full set of BUSCO scores is available at https://blobtoolkit.genomehubs.org/view/xgPatPell1.1/dataset/CAKJPN01/busco.

**Table 2.** Chromosomal pseudomolecules in the genome assembly of *Patella pellucida*, xgPatPell1.1.

INSDC accession	Chromosome	Size (Mb)	GC%
OU795036.1	1	95.03	36.1
OU795037.1	2	91.75	36.3
OU795038.1	3	87.35	36.4
OU795039.1	4	87.16	36.1
OU795040.1	5	79.17	36.3
OU795041.1	6	75.62	36.4
OU795042.1	7	74.06	36.3
OU795043.1	8	59.73	36.5
OU795044.1	9	59.22	36.7
OU795045.1	MT	0.01	32.3
-	Unplaced	2.95	37.3

**Figure 3.**
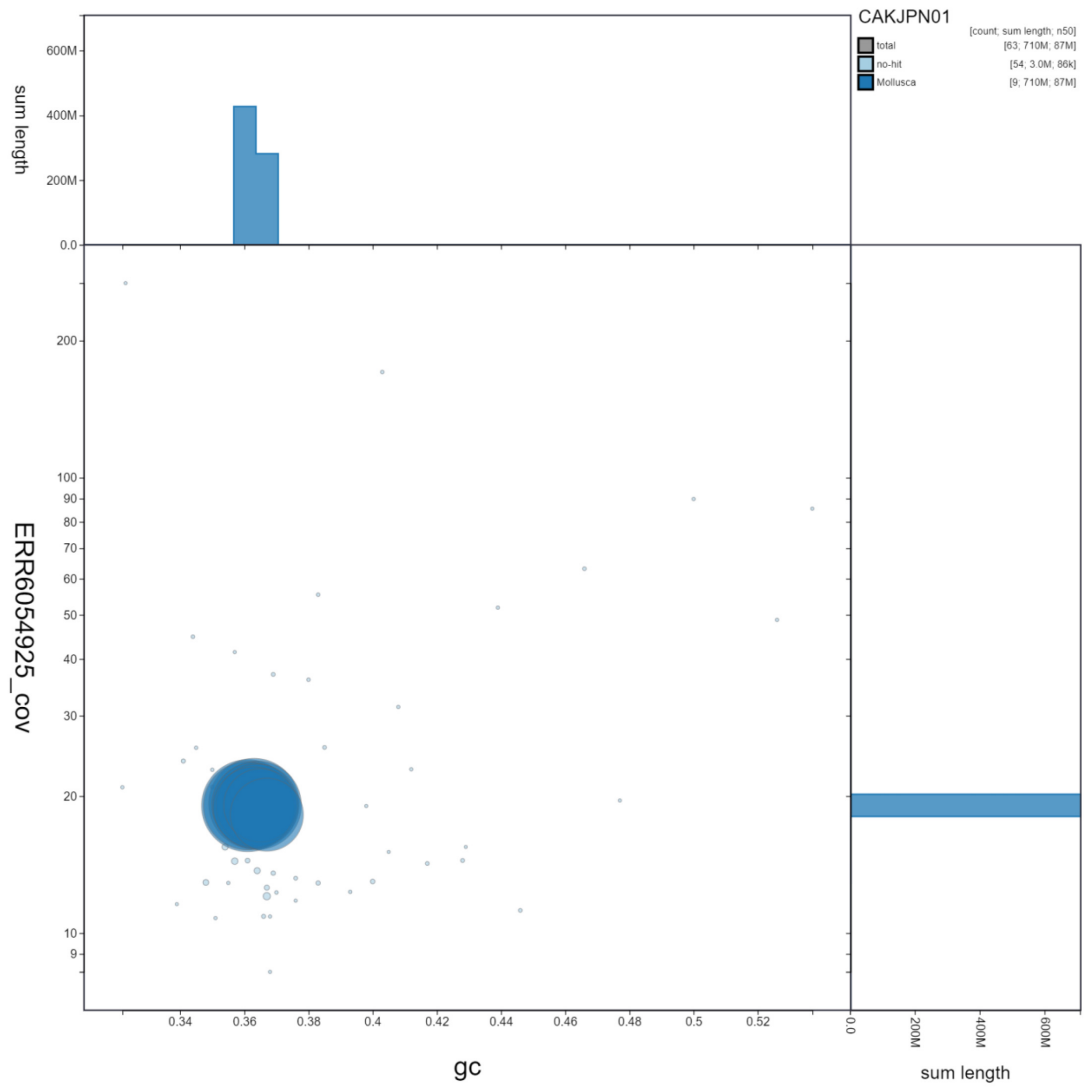
Genome assembly of *Patella pellucida*, xgPatPell1.1. GC coverage. BlobToolKit GC-coverage plot. Scaffolds are coloured by phylum. Circles are sized in proportion to scaffold length. Histograms show the distribution of scaffold length sum along each axis. An interactive version of this figure is available at https://blobtoolkit.genomehubs.org/view/xgPatPell1.1/dataset/CAKJPN01/blob.

**Figure 4.**
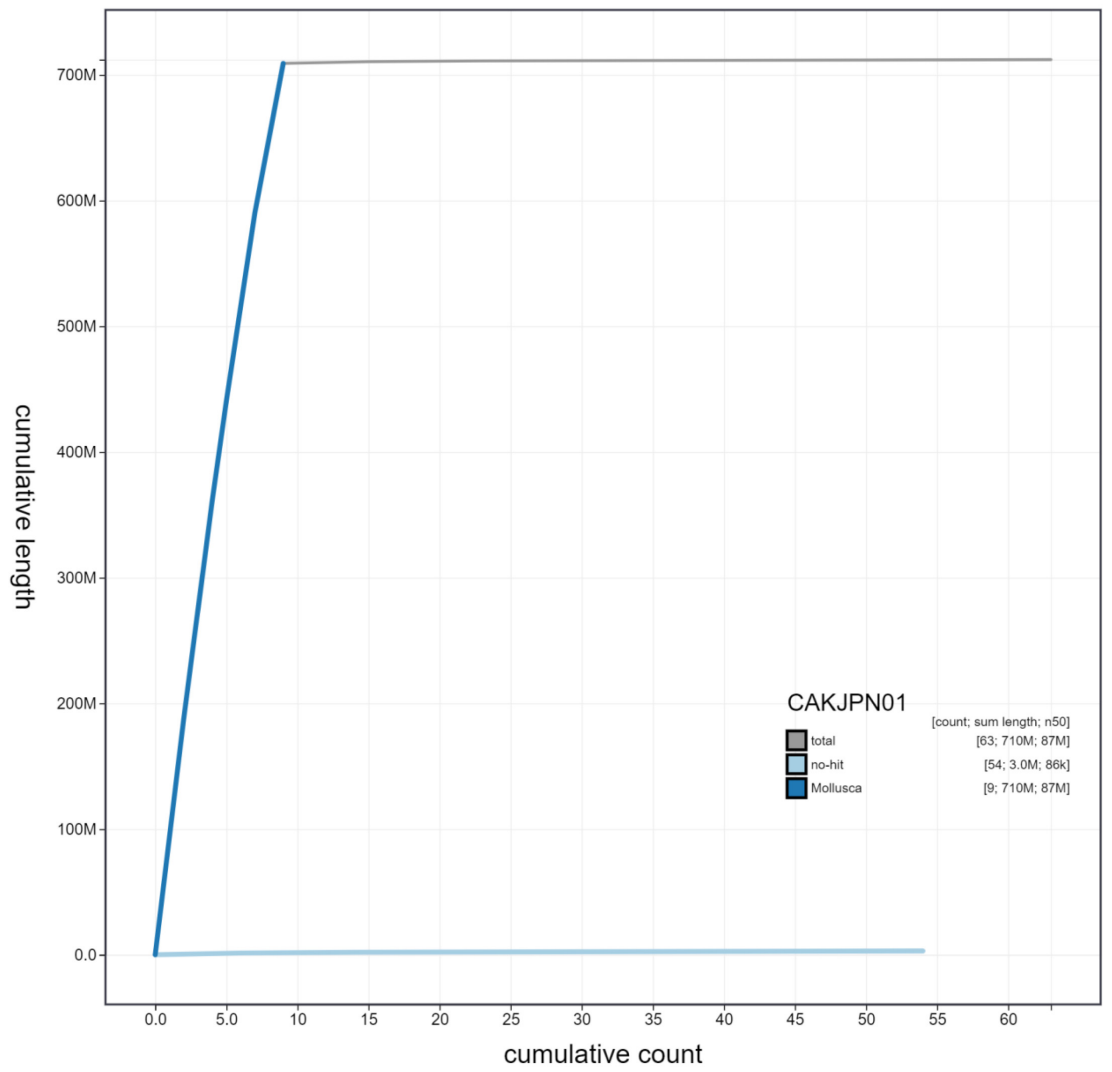
Genome assembly of Patella pellucida, xgPatPell1.1: cumulative sequence. BlobToolKit cumulative sequence plot. The grey line shows cumulative length for all scaffolds. Coloured lines show cumulative lengths of scaffolds assigned to each phylum using the buscogenes taxrule. An interactive version of this figure is available at https://blobtoolkit.genomehubs.org/view/xgPatPell1.1/dataset/CAKJPN01/cumulative.

**Figure 5.**
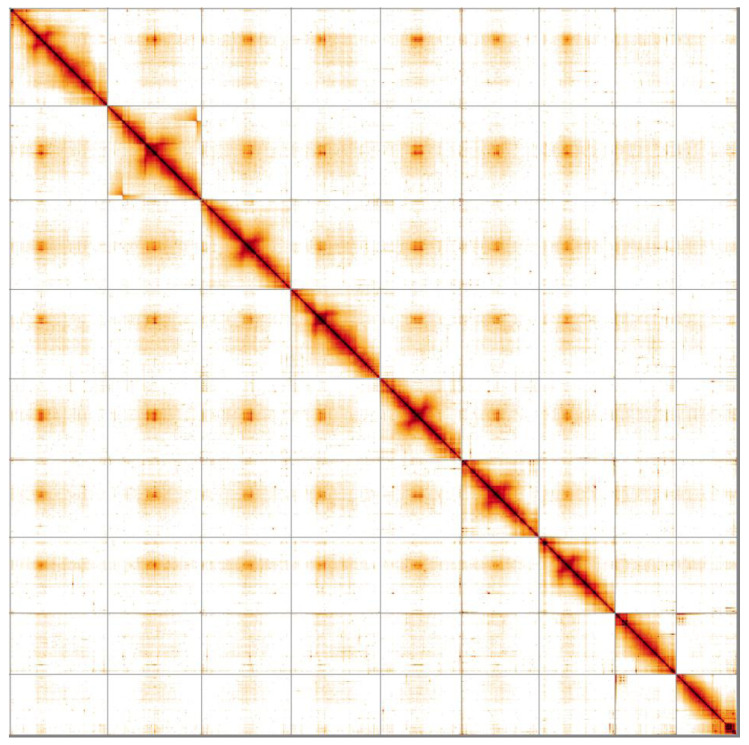
Genome assembly of *Patella pellucida*, xgPatPell1.1: Hi-C contact map. Hi-C contact map of the xgPatPell1.1 assembly, visualised in HiGlass. Chromosomes are shown in size order from left to right and top to bottom.

## Methods

### Sample acquisition and nucleic acid extraction

A total of three *P. pellucida* specimens (xgPatPell1, xgPatPell2, xgPatPell3) were collected from Farland Point, Great Cumbrae, North Ayrshire, UK (latitude 55.74629, longitude -4.911721) by Mara Lawniczak, Wellcome Sanger Institute, by hand from a sheltered rocky shore and boulder cove (old red sandstone bedrock; boulders sandstone and diorite from local dykes). The samples were identified by the same individual and snap-frozen on dry ice.

DNA was extracted at the Tree of Life laboratory, Wellcome Sanger Institute. The xgPatPell1 sample was weighed and dissected on dry ice with the shell removed. Whole organism tissue was cryogenically disrupted to a fine powder using a Covaris cryoPREP Automated Dry Pulveriser, receiving multiple impacts. Fragment size analysis of 0.01–0.5 ng of DNA was then performed using an Agilent FemtoPulse. High molecular weight (HMW) DNA was extracted using the Qiagen MagAttract HMW DNA extraction kit. Low molecular weight DNA was removed from a 200 ng aliquot of extracted DNA using 0.8X AMpure XP purification kit prior to 10X Chromium sequencing; a minimum of 50 ng DNA was submitted for 10X sequencing. HMW DNA was sheared into an average fragment size between 12–20 kb in a Megaruptor 3 system with speed setting 30. Sheared DNA was purified by solid-phase reversible immobilisation using AMPure PB beads with a 1.8X ratio of beads to sample to remove the shorter fragments and concentrate the DNA sample. The concentration of the sheared and purified DNA was assessed using a Nanodrop spectrophotometer and Qubit Fluorometer and Qubit dsDNA High Sensitivity Assay kit. Fragment size distribution was evaluated by running the sample on the FemtoPulse system.

RNA was extracted from whole organism tissue of xgPatPell3 in the Tree of Life Laboratory at the WSI using TRIzol, according to the manufacturer’s instructions. RNA was then eluted in 50 μl RNAse-free water and its concentration RNA assessed using a Nanodrop spectrophotometer and Qubit Fluorometer using the Qubit RNA Broad-Range (BR) Assay kit. Analysis of the integrity of the RNA was done using Agilent RNA 6000 Pico Kit and Eukaryotic Total RNA assay.

### Sequencing

Pacific Biosciences HiFi circular consensus and 10X Genomics read cloud sequencing libraries were constructed according to the manufacturers’ instructions. Poly(A) RNA-Seq libraries were constructed using the NEB Ultra II RNA Library Prep kit. Sequencing was performed by the Scientific Operations core at the Wellcome Sanger Institute on Pacific Biosciences SEQUEL II (HiFi), Illumina HiSeq X (10X) and Illumina HiSeq 4000 (RNA-Seq) instruments. Hi-C data were generated from whole organism tissue of xgPatPell2 using the Arima v2.0 kit and sequenced on a HiSeq X instrument.

### Genome assembly

Assembly was carried out with HiCanu ( Nurk *et al.*, 2020). Haplotypic duplication was identified and removed with purge_dups ( Guan *et al.*, 2020). One round of polishing was performed by aligning 10X Genomics read data to the assembly with longranger align, calling variants with freebayes ( Garrison & Marth, 2012). The assembly was then scaffolded with Hi-C data ( Rao *et al.*, 2014) using SALSA ( Ghurye *et al.*, 2019). The mitochondrial genome was assembled with MitoHiFi ( Uliano-Silva *et al.*, 2021), which performed annotation using MitoFinder ( Allio *et al.*, 2020). The assembly was checked for contamination and corrected using the gEVAL system ( Chow *et al.*, 2016) as described previously ( Howe *et al.*, 2021). Manual curation ( Howe *et al.*, 2021) was performed using gEVAL, HiGlass ( Kerpedjiev *et al.*, 2018) and Pretext. The genome was analysed within the BlobToolKit environment ( Challis *et al.*, 2020). Table 3 contains a list of all software tool versions used, where appropriate.

**Table 3.** Software tools used.

Software tool	Version	Source
HiCanu	2.1	Nurk *et al.*, 2020
purge_dups	1.2.3	Guan *et al.*, 2020
SALSA2	2.2	Ghurye *et al.*, 2019
longranger align	2.2.2	https://support.10xgenomics.com/genome-exome/ software/pipelines/latest/advanced/other-pipelines
freebayes	1.3.1-17- gaa2ace8	Garrison & Marth, 2012
MitoHiFi	2	https://github.com/marcelauliano/MitoHiFi
gEVAL	N/A	Chow *et al.*, 2016
HiGlass	1.11.6	Kerpedjiev *et al.*, 2018
PretextView	0.2.x	https://github.com/wtsi-hpag/PretextView
BlobToolKit	2.6.4	Challis *et al.*, 2020

### Ethics/compliance issues

The materials that have contributed to this genome note have been supplied by a Darwin Tree of Life Partner. The submission of materials by a Darwin Tree of Life Partner is subject to the Darwin Tree of Life Project Sampling Code of Practice. By agreeing with and signing up to the Sampling Code of Practice, the Darwin Tree of Life Partner agrees they will meet the legal and ethical requirements and standards set out within this document in respect of all samples acquired for, and supplied to, the Darwin Tree of Life Project. Each transfer of samples is further undertaken according to a Research Collaboration Agreement or Material Transfer Agreement entered into by the Darwin Tree of Life Partner, Genome Research Limited (operating as the Wellcome Sanger Institute), and in some circumstances other Darwin Tree of Life collaborators.

## Data availability

European Nucleotide Archive: Patella pellucida (blue-rayed limpet). Accession number PRJEB45187; https://identifiers.org/ena.embl/PRJEB45187.

The genome sequence is released openly for reuse. The *P. pellucida* genome sequencing initiative is part of the Darwin Tree of Life (DToL) project. All raw sequence data and the assembly have been deposited in INSDC databases. The genome will be annotated with the RNA-Seq data and presented through the Ensembl pipeline at the European Bioinformatics Institute. Raw data and assembly accession identifiers are reported in Table 1.

## Author information

Members of the Darwin Tree of Life Barcoding collective are listed here: https://doi.org/10.5281/zenodo.5744972.

Members of the Wellcome Sanger Institute Tree of Life programme are listed here: https://doi.org/10.5281/zenodo.6125027.

Members of Wellcome Sanger Institute Scientific Operations: DNA Pipelines collective are listed here: https://doi.org/10.5281/zenodo.5746904.

Members of the Tree of Life Core Informatics collective are listed here: https://doi.org/10.5281/zenodo.6125046.

Members of the Darwin Tree of Life Consortium are listed here: https://doi.org/10.5281/zenodo.5638618.
