# Supplementary material for: The genome sequence of the blue-rayed limpet, Patella pellucida Linnaeus, 1758
Source: Wellcome Open Res. 2022 Apr 4;7:126. [Version 1] doi: 10.12688/wellcomeopenres.17825.1 (PMC9975397; doi:10.12688/wellcomeopenres.17825.1)
Supplement: Peer review history

# Peer review: Reviewer response for version 1

**DOI:** 10.21956/wellcomeopenres.19735.r53596

**Published:** 2023-01-11

**Authors:** Marco Gerdol

**Affiliations:** 1 Department of Life Sciences, University of Trieste, Trieste, Italy

This genome report is a welcome addition to the growing amount of genomic resources available for the phylum Mollusca. In particular, the blue-rayed limpet is a common marine species of broad interest, and this is the first high quality genome to be generated for Patellogastropoda, many years after the publication of the Lottia gigantea genome, which has been a reference for the scientific community for a very long time, despite the limitations linked to its fragmentation and relatively poor quality.

 The genome assembly has been carried out using gold-standard methodologies and I don't see any significant issue, as all the data reported is scientifically sound. I only have a minor comment concerning the brief mention of structural variation made by the authors, with a large inversion found in chromosome 2. Molluscan genome notoriously display high heterozygosity levels and structural variation might be much more widespread than originally thought, even though, in this specific case, all the metrics seem to suggest that the hemizygous portion of the genome was not particularly relevant (assembly size was only reduced by 1.72% after manual curation). I think a missing bit of information here is the heterozygosity rate calculated based on k-mer graphs, as this could be a useful additional information. Also, the authors may consider adding a k-mer spectrum analysis with Merqury (this is not essential, but could be worth).

Are sufficient details of methods and materials provided to allow replication by others?

Yes

Is the rationale for creating the dataset(s) clearly described?

Yes

Are the datasets clearly presented in a useable and accessible format?

Yes

Are the protocols appropriate and is the work technically sound?

Yes

Reviewer Expertise:

Comparative genomics

I confirm that I have read this submission and believe that I have an appropriate level of expertise to confirm that it is of an acceptable scientific standard.

# Peer review: Reviewer response for version 1

**DOI:** 10.21956/wellcomeopenres.19735.r53599

**Published:** 2022-12-13

**Authors:** Coen Adema

**Affiliations:** 1 Department of Biology, University of New Mexico, Albuquerque, NM, USA

Application of RNAseq, Pacbio and Hi-C to Patella lucida yields valuable addition to genome sequence data of molluscs. The analysis provides an indication of chromosome number in basal gastropods by showing hi-C pseudochromosomes. The discussion of the molluscan reference set for BUSCO is useful. Recommend to include rationale for selecting this particular species for genome sequencing. Useful to include taxonomy; i.e. was it strategically chosen as representative of basal or derived group to inform on molluscan biology? Is it a particular pest or food source? Is it randomly chosen as part of overall bio-inventory effort?

 Fig 1, put numbers in figure to indicate actual specimen(s) used.

 Include brief mention of outcome of mitogenome assembly. Was the mitogenome confirmed to be complete based on gene complement?

 Mitogenome sequence accession record would have increased usability with annotation.

 Clarify whether the inversion reported for sister chromatids of CHR2 is interpreted as assembly artefact or has biological relevance. Do termini of the inversion interrupt coding sequences or locate to intergenic regions.

Are sufficient details of methods and materials provided to allow replication by others?

Yes

Is the rationale for creating the dataset(s) clearly described?

No

Are the datasets clearly presented in a useable and accessible format?

Partly

Are the protocols appropriate and is the work technically sound?

Yes

Reviewer Expertise:

Molluscan immuno-genomics

I confirm that I have read this submission and believe that I have an appropriate level of expertise to confirm that it is of an acceptable scientific standard.

# Peer review: Reviewer response for version 1

**DOI:** 10.21956/wellcomeopenres.19735.r50657

**Published:** 2022-06-06

**Authors:** Masa-aki Yoshida

**Affiliations:** 1 Oki Marine Biological Station, Faculty of Life and Environmental Sciences, Shimane University, Matsue, Japan

This paper by Lawniczak *et al. *combines data from PacBio, 10x, and Hi-C to decode the genome of *Patella pellucida* (the blue-rayed limpet) in sufficient quality to meet the criteria of the high quality genome report. By the Darwin Tree of Life's standard for publishing genome, this genome data was shown to make the data widely available to a variety of researchers. While the report is commendable for covering the necessary and sufficient content for a genome report, the following minor points should be corrected.

1. It is difficult to accept the top9 scaffolds as “chromosome”, although they are certainly long enough. It would be better to do linkage mapping and comparison with closely related chromosomes, or else to keep them as long scaffolds.
2. I agree that the mollusca_odb10 reference set gives assembly an unfairly low value. I think that the BUSCO score shown in Table 1 and Figure 1 should be that of metazoa_ odb 10, which would be more appropriate for comparison with the current genome of many species.
3. It is not clear what is being compared to what in the following statement. "Chromosome 2 contains a large inversion between sister chromatids at approximately 15.43-87.97 Mb. The inversion spans the majority of the 91.75 Mb chromosome.” Does this mean that there is a common sequence between chromosomes 1 and 2 that is causing the inversion? Or does it indicate an inversion between haplotypes? I think a more detailed explanation is needed.
4. The authors speculate that the stripes on the shell are present to mimic toxic gastropods. To what species is it supposed to mimic? It would be better to be specific so that later ones can be verified.

Are sufficient details of methods and materials provided to allow replication by others?

Yes

Is the rationale for creating the dataset(s) clearly described?

Yes

Are the datasets clearly presented in a useable and accessible format?

Yes

Are the protocols appropriate and is the work technically sound?

Yes

Reviewer Expertise:

Evolutionary genomics

I confirm that I have read this submission and believe that I have an appropriate level of expertise to confirm that it is of an acceptable scientific standard.
